# Effect of Three Colostrum Diets on Passive Transfer of Immunity and Preweaning Health in Calves on a California Dairy following Colostrum Management Training

**DOI:** 10.1155/2014/698741

**Published:** 2014-04-16

**Authors:** Deniece R. Williams, Patrick Pithua, Angel Garcia, John Champagne, Deborah M. Haines, Sharif S. Aly

**Affiliations:** ^1^Veterinary Medicine Teaching and Research Center, School of Veterinary Medicine, University of California, Davis, 18830 Road 112, Tulare, CA 93274, USA; ^2^Department of Veterinary Medicine & Surgery, Veterinary Medicine Teaching Hospital, College of Veterinary Medicine, University of Missouri, 900 E. Campus Drive, Columbia, MO 65211, USA; ^3^Departamento de Producción y Sanidad Animal, Facultad de Veterinaria, Universidad CEU Cardenal Herrera, Moncada, 46113 Valencia, Spain; ^4^Department of Veterinary Microbiology, Western College of Veterinary Medicine, University of Saskatchewan, Saskatoon, SK, Canada S7N 5B4; ^5^The Saskatoon Colostrum Co. Ltd., 30 Molaro Pl, Saskatoon, SK, Canada S7K 6A2; ^6^Department of Population Health and Reproduction, School of Veterinary Medicine, University of California, One Shields Avenue, Davis, CA 95616, USA

## Abstract

Following colostrum management training, a randomized field trial was conducted on a California dairy to determine the effect of supplementing pooled colostrum with either colostrum-derived replacer (CDR) or second-milking colostrum (transition milk) on failure of passive transfer (FPT) and preweaning morbidity risks. A total of 166 calves were randomly assigned to 4L first-milking pooled colostrum (treatment 1), 2L first-milking pooled colostrum and 2L of CDR (treatment 2), or 2L first-milking pooled colostrum and 2L second-milking pooled colostrum (treatment 3). Mean 24-hour serum TP and IgG for treatments 2 (TP 5.2 g/dL, IgG 15.9 g/L) and 3 (TP 5.4 g/dL, IgG 18.3 g/L) did not statistically differ but were significantly lower than for treatment 1 (TP 5.9 g/dL, IgG 24.6 g/L). Risk of FPT did not differ for treatments 1, 2, and 3 (0.0%, 9.3%, and 1.9%, resp.). Similarly, the preweaning risk of diarrhea (81.0%, 92.5%, and 87.0%, resp.) or pneumonia (6.9%, 13.2%, and 18.5%, resp.) did not differ between treatments. Feeding 4L first-milking pooled colostrum resulted in adequate passive transfer. When first-milking pooled colostrum quantity is inadequate, CDR or second-milking pooled colostrum can be used to supplement the required colostrum volume and IgG mass without adversely affecting the risks of FPT or preweaning diarrhea and pneumonia.

## 1. Introduction

Timely ingestion of high quality colostrum is one of the most important factors affecting both short and long term calf performance. Calves that ingest >2 L of high quality colostrum or that have higher levels of serum IgG in the first week of life have lower morbidity and mortality than calves with lower serum IgG levels or that ingest insufficient quantities of high quality colostrum. Adequate passive transfer of immunity has been associated with lower preweaning veterinary costs, improved weight gain, and increased milk production and longevity in the milking herd [1–4]. Current recommendations to reduce the incidence of failure of passive transfer of immunity (FPT), defined as serum IgG < 10 mg/mL in the first 1 to 7 days of life, include feeding 3 to 4 L of colostrum containing a minimum of 50 mg/mL IgG within the first 1 to 2 hours of birth [3, 5–8]. Additionally, studies have shown that colostrum with bacterial counts <100,000 cfu/mL has a superior apparent efficiency of absorption (AEA) of immunoglobulins than colostrum with higher bacterial counts, when fed to calves [5, 9]. Despite these recommendations, a recent survey of US dairies reported that, among those tested, 40.7% of dairies had at least one calf with FPT and that 19.2% of all calves fail to achieve adequate passive transfer of immunity, with calves in the Western USA experiencing a higher FPT rate than calves in the Eastern USA [10, 11]. Understanding the underlying factors contributing to FPT on dairies is essential to reduce the occurrence of this condition and to improve short term calf performance and long term dairy productivity.

A management practice contributing to FPT may be pooling colostrum. Based on a 2007 National Animal Health Monitoring survey of US dairy herds, Beam and others estimated that calves fed pooled colostrum were more than twice as likely to have FPT as calves fed colostrum from an individual cow [10, 11]. In addition, a recent study conducted on a California dairy showed that calves fed pooled colostrum with a low IgG concentration (21.1 g/L) had significantly lower 24-hour serum total protein and IgG concentrations and a significantly higher risk of FPT compared to calves fed colostrum-derived replacer (CDR) [12]. The California study also showed that both the colostrum IgG concentration and the IgG mass fed were significantly higher in the CDR compared to the pooled colostrum [12]. Pooling can maintain an adequate supply of colostrum but may result in lower quality due to dilution of IgG, as cows producing higher volumes often contribute colostrum with a lower concentration of immunoglobulins [3]. Immunoglobulins are present at high concentrations in colostrum, the initial secretion from the mammary gland following parturition. While the levels of immunoglobulins decrease rapidly with each milking, they remain at higher levels than found in milk for 5–7 days after calving, as the cow transitions to production of normal milk [13–18]. Intentional or accidental addition of colostrum from the second or later milkings (also known as transition milk), to the colostrum pool can, therefore, lead to dilution of the IgG concentration of the pooled colostrum. Furthermore, it is common practice on California dairies to house recently calved cows in the hospital pen. As such, sick cows, newly admitted into the hospital pen, may be misidentified in the parlor, allowing milk from nonfresh cows to be harvested into the colostrum supply, further contributing to dilution of the IgG in the colostrum pool. In a recent national study of colostrum quality in the USA, only 25.6% of pooled colostrum samples met industry standards for both IgG concentration and bacterial count compared with 41.2% of nonpooled samples [19]. Pooling colostrum also increases the risk of exposure to infectious disease organisms, such as* Mycobacterium avium* subsp.* paratuberculosis*, bovine leukosis virus, and bovine viral diarrhea virus [20]. Despite the recognized biosecurity risks and negative effect on passive transfer of immunity associated with feeding pooled colostrum, calves in 16% of US herds with <100 cows and approximately 57% of herds with >500 cows continue to be fed pooled colostrum [11].

A second, related management factor contributing to FPT on dairies is a shortage in colostrum supply and, in response, commercial colostrum supplements and replacer products have been developed from bovine plasma, serum, or colostrum [20]. Results of studies evaluating the efficacy of these products on serum IgG concentrations, rates of FPT, and preweaning health outcomes in calves are mixed. Colostrum replacers and supplements derived from plasma or serum that provided higher doses of IgG (>170 g IgG) were shown to prevent FPT; however, those with lower IgG doses were not [21–26]. Similarly, colostrum-derived replacers with high doses of IgG (>170 g IgG) were shown to prevent FPT; however, some but not all colostrum-derived replacers and supplements with lower doses of IgG also prevented FPT [8, 12, 25, 27–31]. Studies of plasma-derived replacers have shown either no difference or an increased risk of health events in calves compared to colostrum, whereas studies of colostrum-derived replacers have shown both a decreased and increased risk of health events in calves compared to colostrum [23, 25, 26, 32]. Feeding pooled colostrum in conjunction with a CDR has not been evaluated and could be an effective approach to address colostrum shortages on dairies.

The first objective of this study was to evaluate the effect of implementing colostrum management training regarding the collection and pooling of colostrum on the IgG concentration in pooled colostrum, on a dairy previously shown to have low quality, pooled colostrum [12]. For the purposes of this study, colostrum is defined as the lacteal secretion produced for the first 5 days of lactation [13, 15–17]; however, only first- and second-milking colostrum pools were used based on the milking number from which they were obtained. The second objective was to conduct a randomized field trial to compare the effect of feeding first-milking pooled colostrum or first-milking pooled colostrum followed by either a reconstituted CDR or second-milking pooled colostrum on the risk of FPT, morbidity, and mortality in preweaning Jersey and Holstein calves. Results of this study will help veterinarians and dairy producers more effectively manage colostrum supply, ultimately improving calf performance on dairies.

## 2. Materials and Methods

### 2.1. Study Herd and Experimental Design

This study was approved by the University of California at Davis Institutional Animal Care and Use Committee (approval date 2/22/2012). The trial was conducted on a Central California Holstein and Jersey dairy in Tulare County which milked 3,600 cows and fed unpasteurized pooled colostrum to calves. The dairy was selected because of its large size, records availability through the California Dairy Herd Improvement Association's milk testing program, and the owner's interest and willingness to participate in the study.

In a previous study, pooled colostrum collected from the study herd had lower IgG concentrations (mean 21.1 g/L, SD 9.76) than the reported average for Southwestern US dairies (64.3 g/L, SE = 2.9) which may explain the high proportion of FPT in the dairy's calves [12, 19]. Hence, the herd provided a unique opportunity for colostrum management training to improve the IgG concentration of pooled colostrum.

### 2.2. Pretrial Training Period

#### 2.2.1. Colostrum Management Training

Between November 2011 and January 2012, training was provided to the dairy staff by the study veterinarians to prevent misidentification of fresh and sick cows in the hospital pen and to eliminate mixing first- and second-milking colostrum within the same pool. All cows were milked twice a day and the dairy's protocols regarding timing of milking or parlor hygiene remained unaltered. After calving and prior to the first milking, maternity pen staff used a degradable color wax stick to mark each cow's hind limb with a line from above the hock to below the tarsal joint. Only cows with a line contributed to the first-milking pooled colostrum supply. After the first milking, a single cross hatch was placed over this line by the milking staff. Only cows with the single cross hatch supplied the second-milking colostrum. After the second milking, another cross hatch was placed to indicate that these cows were excluded from further colostrum collection. First- and second-milking pooled colostrum were collected into receiver containers that had been permanently marked with the labels “1” and “2”, respectively.

#### 2.2.2. Baseline Estimation of Herd-Level Colostrum IgG Concentration

Prior to initiation of the current trial and while colostrum management training was ongoing, colostrum samples were collected between November 2011 and January 2012 to evaluate the effect of the colostrum management training on baseline IgG concentration of the colostrum. Herd personnel collected a colostrum sample (50 mL) from the first- and second-milking colostrum containers after each milking. These colostrum samples were frozen on the dairy at −20°C and were transported on a weekly basis to the University of California at Davis, Veterinary Medicine Teaching and Research Center (VMTRC, Tulare, CA). The samples were stored at −80°C until the end of the pretrial period, at which point all samples were sent to the testing laboratory (The Saskatoon Colostrum Co. Ltd., Saskatoon, Canada) for estimation of IgG (g/L) concentration using radial immunodiffusion. 

### 2.3. Trial Period

#### 2.3.1. Estimation of Sample Size

Sample size for a significant difference in 24-hour serum IgG among the three groups of calves estimated for multiple comparisons using the Tukey-Kramer (Pairwise) method, at a power of 95%, an alpha of 0.05, a minimal detectable difference of 1 unit, and a SD = 2 resulted in a sample size of 50 calves per group. Adding an additional 20% to account for attrition, the total sample size of 60 calves per group was estimated.

#### 2.3.2. Colostrum and Feeding Treatments

Cows within the last 2 weeks of pregnancy were monitored by trained dairy staff and moved to a group maternity pen upon showing signs of impending parturition. Calves were separated from the dam as soon as possible after birth to prevent suckling and moved to a group pen where they were ear-tagged, had their navels dipped with 7% iodine tincture, and were fed colostrum. Jersey heifer calves, Holstein heifer calves, and Holstein bull calves were enrolled between February and March, 2012. Jersey bull calves, all cross-breeds, and calves born between 5 pm and 9 pm, Monday through Thursday, and between 5 pm Friday and 9 pm Sunday were excluded from enrollment into the study.

Calves were randomly assigned to one of three treatments using a list of random numbers pregenerated in Excel 2010 (Microsoft, Redmond, WA). The randomization list was sealed with opaque adhesive notes that were sequentially removed only at each calf's birth to reveal treatment assignment. In treatment 1, calves were fed 4 L of raw, first-milking pooled colostrum. In treatment 2, calves were fed 2 L of raw, first-milking pooled colostrum followed immediately by 2 L of CDR (Calf's Choice Total HiCal, Alta Genetics Inc.). In treatment 3, calves were fed 2 L of raw, first-milking pooled colostrum followed immediately by 2 L of raw, second-milking pooled colostrum.

Colostrum was harvested and stored as described for the pretrial period. First- and second-milking pooled colostrum were divided into individual plastic nipple bottles, stored at 4°C until feeding, and warmed prior to feeding. Calves were fed colostrum harvested on the day of their birth. The CDR powder was reconstituted according to the manufacturer's instruction by dissolving 700 g of the powder in 1.25 L of warm water (46–57°C), ensuring a solvent that provided 100 g of IgG per dose. Holstein calves that did not suckle after multiple attempts or failed to ingest the entire volume within 3 hours were fed the remaining volume via an esophageal tube feeder. Jersey heifer calves that did not voluntarily ingest 4 L of colostrum were instead tube-fed a volume of 3 L of their assigned colostrum treatment.

#### 2.3.3. Preweaning Calf Management

All study calves were housed in individual, elevated wooden hutches, and given free choice water and starter grain until weaning at approximately 60 to 75 days of age. Calves were fed 2 L of pasteurized waste milk supplemented with milk replacer containing 20% protein and 20% fat, twice a day. In April 2012, the dairy's management implemented changes to the calf feeding program. Changes affected the type and quantity of milk fed and the number of feedings per day. The youngest study calf affected by these changes was 20 days old.

### 2.4. Data Collection and Laboratory Analysis of Samples

Information recorded for each calf included herd identification number, sex, breed, birthdate, dam identification, birth weight (kg), time to separation from dam (hrs), and time to colostrum feeding (hrs).

Immediately before and 24 hours after colostrum was fed, a blood sample (5 mL) from each calf was collected by jugular venipuncture, allowed to clot, and centrifuged (Model E8, LW Scientific, Lawrenceville, GA) at 3300 rpm within 30 minutes of collection. Serum samples were harvested, divided into two aliquots, and labeled with the calf ID and whether the sample was collected before or after feeding colostrum.

A sample of pooled colostrum (5 mL) fed to each calf was collected before feeding and labeled with calf ID and milking number. All samples were frozen at −20°C, transported on ice to the VMTRC, and stored at −80°C until shipment to the testing laboratory (The Saskatoon Colostrum Co. Ltd., Saskatoon, Canada) for further analyses. Serum samples were analyzed for total protein (g/dL) by digital refractometer. The IgG (g/L) of the colostrum and serum samples were analyzed using radial immunodiffusion [27]. All laboratory personnel were blinded to treatment.

#### 2.4.1. Health Monitoring

Each calf was monitored twice daily by dairy staff and at least once daily by study personnel for signs of illness including lethargy, weakness, decreased appetite, dehydration, fever, abnormal fecal consistency, cough, increased respiratory rate, ocular or nasal discharge, drooping ears, and head tilt. All health events and medical treatments prescribed by the herd veterinarian were recorded on waterproof cards hanging on each calf hutch. Data on the health events and treatments for each calf was subsequently transferred daily to a standard database by study personnel until the end of follow-up for each calf at approximately 53 (±3) days of age. All personnel monitoring the calves were blinded to treatment.

The preweaning morbidity events of interest in this study were diarrhea and pneumonia. To ensure consistency, the Wisconsin Calf Health Scoring Chart was modified and used as a guide in the diagnosis of health events [33]. Diarrhea was defined as a calf passing abnormal, watery feces with a foul odor (score 2 or 3). Pneumonia was defined as a calf displaying signs of spontaneous cough and increased respiratory rate, with or without ocular and/or nasal discharge or otitis (score 2 or 3), except no attempt was made to induce a cough. A health event that occurred more than seven days following recovery from a previous health event was considered a new incident. Health events occurring within seven days of a previous health event with similar clinical signs were considered the same incident.

### 2.5. Statistical Analysis

All statistical analyses were performed using standard statistical software (IBM SPSS Statistics, version 22.0.0, Armonk, NY, IBM Corp.) and were considered significant if the *P* value was <0.05. Mean ± standard error of the mean (SE) pooled colostrum IgG concentrations for the study pretrial and trial periods were calculated and compared using a one-way ANOVA. For each treatment in the study, categorical baseline characteristics (sex, breed, time to separation from dam, and time to colostrum feeding) were compared using a Pearson chi-square test or Fischer's exact test. Continuous baseline characteristics (birth weight, precolostrum serum total protein, precolostrum serum IgG, first-milking colostrum IgG, and total IgG fed) were compared among treatments using one-way ANOVA. The AEA for each calf was calculated using the following formula:

(1) $$\begin{array}{c} \text{AEA}=\frac{\text{Serum}\;\text{IgG}\;\left(\text{g}/\text{L}\right)\times \text{Serum}\;\text{volume}\;\left(\text{L}\right)}{\text{IgG}\;\text{Intake}\;\left(\text{g}\right)} \end{array}$$

with plasma volume estimated as 9.9% and 9.7% of birth weight for Holsteins and Jerseys, respectively [34]. Means of the AEA of IgG (%) for the treatments were compared using a one-way ANOVA. Proportions of calves with FPT (defined as 24-hour postfeeding serum IgG < 10 g/L), diarrhea, and pneumonia among the treatments were compared using a *Z*-test with the alpha level adjusted for multiple comparisons using the Bonferroni method.

Two separate Cox proportional hazard models were used to estimate and compare the preweaning hazards for both diarrhea and pneumonia among treatments. Follow-up time for all calves occurred from enrollment until just prior to weaning (53 ± 3 days of age). The proportional hazards assumption was evaluated graphically and using the Schoenfeld residuals. Violation in the proportional hazards assumption was addressed by estimating hazard ratios using an extended Cox model with time-dependent covariates. Variables considered in the models were treatment, sex, and breed. With the exception of treatment, which was forced into each model, all variables were entered into the Cox models using a backwards stepwise elimination procedure. Variables were selected for removal if the *P* value for the change in the log-likelihood ratio was greater than 0.10.

## 3. Results

### 3.1. Effect of Colostrum Management Training on Pooled Colostrum Quality

In the pretrial period, a total of 56 first-milking pooled colostrum and 66 second-milking pooled colostrum samples were analyzed. The mean (±SE) IgG concentrations in these samples were 56.3 g/L (±2.69) and 18.3 g/L (±1.72), respectively. In the trial, a total of 166 first-milking pooled colostrum and 53 second-milking pooled colostrum samples were tested. The mean (±SE) IgG concentrations in these samples were 65.1 g/L (±1.25) and 25.8 g/L (±1.81), respectively. The first-milking pooled colostrum from the trial period had a mean IgG concentration that was significantly higher than the first-milking pooled colostrum in the pretrial period (*P* = 0.003) and the second-milking pooled colostrum in both the pretrial and trial periods (*P* < 0.001). Similarly, the mean IgG concentration of the pretrial, first-milking pooled colostrum was significantly higher than the second-milking pooled colostrum from the pretrial and trial periods (*P* = 0.003). No statistical difference was noted between the mean IgG concentration of the second-milking pooled colostrum in the pretrial and trial periods (*P* = 0.059); however this comparison had low statistical power because of the small number of samples tested.

### 3.2. Effect of Colostrum Type on Passive Transfer of Immunity, Preweaning Health, and Survival

A total of 187 calves were enrolled in the study. Of these, 166 calves comprising 57 Holstein heifers, 85 Holstein bulls, and 24 Jersey heifers remained eligible for statistical analysis. Of those enrolled, 58, 54, and 54 calves were allocated to treatments 1, 2, and 3, respectively. Of the 21 calves excluded from the analysis, 16 had precolostrum serum IgG concentrations greater than 2.3 g/L (5, 7, and 4 calves from treatments 1, 2, and 3, resp.) due to possible colostrum ingestion prior to sampling [35]. The remaining 5 calves that were excluded consisted of four calves that died within 48 hours from atresia coli, which was not apparent at birth (treatment 2: one calf and treatment 3: three calves), and one calf (treatment 2) that had no 24-hour serum sample taken. An additional calf (treatment 2) was euthanized by the dairy at 9 days of age due to a leg injury and was excluded from analysis of health events. A second-milking colostrum sample for one calf was not collected; therefore, comparison of second-milking colostrum IgG concentration, total IgG fed, and AEA among treatments was based on 165 calves.

Of the baseline characteristics tested, only total IgG (g) fed was significantly different among treatments, with calves fed only first-milking pooled colostrum (treatment 1) having the highest and calves fed first- and second-milking pooled colostrum having the lowest (treatment 3) mean total IgG fed (Table 1). Calves that received only first-milking pooled colostrum had 24-hour serum TP and IgG that was significantly higher than calves in the other treatments (*P* < 0.001); however, no difference was noted between the calves fed first-milking pooled colostrum followed by either CDR or second-milking pooled colostrum (Table 2). Calves that received first-milking pooled colostrum followed by CDR (treatment 2) had a significantly lower AEA than calves that received either first-milking pooled colostrum alone or a mixture of first- and second-milking pooled colostrum (*P* < 0.001, Table 2). Although numerical differences existed, no statistical difference was noted in the risk of FPT as defined by serum IgG < 10 g/L (*P* ≥ 0.05) among the treatments (Table 2). No statistical difference (*P* ≥ 0.05) was noted among treatments for the risk of preweaning diarrhea (treatment 1: 81.0%, *n* = 58, SE = 0.052, treatment 2: 92.5%, *n* = 53, SE = 0.037, and treatment 3: 87.0%, *n* = 54, SE = 0.046) or pneumonia (treatment 1: 6.9%, *n* = 58, SE = 0.034, treatment 2: 13.2%, *n* = 53, SE = 0.047, and treatment 3: 18.5%, *n* = 54, SE = 0.053). Two calves (treatment 2) died from diarrhea. No other deaths due to infectious disease occurred during the study period.

The final Cox model for diarrhea was adjusted for breed where Jersey calves' hazard rate ratio (HRR) for diarrhea was 2.13 times that of Holsteins (95% CI (1.35, 3.35), *P* = 0.001). In comparison, the best-fitting model for pneumonia was adjusted for gender; however, the hazard for bull compared to heifer calves was not significant (HRR = 0.39, 95% CI (0.151, 1.01), *P* = 0.39). In both models, the treatment variable violated the proportional hazard assumption and a treatment-time interaction term was forced into the model. The hazard for diarrhea was not significant for treatment 2 or 3 (HRR = 1.03, 95% CI (0.997, 1.07), *P* = 0.074; HRR = 1.02, 95% CI (0.98, 1.06), *P* = 0.332, resp.) compared to treatment 1. Similarly, the hazards for pneumonia for treatment 2 or 3 were not significant (HRR = 1.04, 95% CI (0.99, 1.09), *P* = 0.100; HRR = 1.04, 95% CI (0.99, 1.09), *P* = 0.114, resp.) compared to treatment 1.

## 4. Discussion

Feeding 4 L of first-milking pooled colostrum to calves ensured adequate passive transfer of immunity. However, dairies that properly manage the quality of pooled colostrum may, at times, experience a shortage of first-milking colostrum. The current study is the first to investigate the alternatives of feeding 2 L of first-milking pooled colostrum followed by either 2 L of CDR or 2 L of second-milking pooled colostrum. Although mean 24-hour serum IgG levels were significantly lower in calves fed either alternative, mean IgG levels in all groups were excellent [36] and the risks of FPT and preweaning diarrhea or pneumonia were not significantly different from calves fed 4 L of first-milking pooled colostrum. The lack of significant differences in FPT or preweaning morbidity between treatments could have been due to a small sample size or the fact that all treatments delivered a mass of IgG that met or exceeded the current industry recommendation of feeding 150–200 g IgG in 3-4 L of colostrum [3, 5–8] and far exceeded the historical recommendation of 100 g IgG in 2 L of colostrum [37, 38].

In addition, feeding first-milking pooled colostrum followed by either CDR or second-milking pooled colostrum did not result in an increased hazard for either diarrhea or pneumonia compared to feeding first-milking pooled colostrum alone. However, these results should be interpreted with caution as it is possible that the diet changes implemented by the dairy management during the follow-up period affected the risk of diarrhea or pneumonia for the calves. Improved nutrient intake has been associated with improved immune function [39]. Therefore, the diet changes may have provided protection against diarrhea or pneumonia, biasing the results by decreasing disease incidence. However, at the onset of the diet changes, the youngest calf was 20 days of age, which is older than the mean age of onset reported for the major causes of preweaning calf diarrhea [40]. Therefore, it is possible that the calves had passed the period of highest risk for diarrhea prior to the onset of diet changes such that the hazard results for diarrhea were unaffected. Additionally, survival analysis for diarrhea in a subset of the data for calves from birth to 20 days of age showed a nonsignificant hazard for diarrhea (data not shown), which is in agreement with the results of the Cox extended model based on the entire follow-up period.

Feeding high quality colostrum is an important management tool for ensuring healthy and productive calves. Results from this study indicated that dairies with management practices similar to the study herd could significantly improve the IgG concentration of first-milking pooled colostrum by implementing colostrum management training to prevent misclassification of fresh cows. As with many large California dairies, the study dairy housed fresh and sick cows in the same pen. Before initiation of the colostrum management training in this herd, there was no formal method to distinguish the fresh cows in the milking parlor. Thus, colostrum from the third or later milkings and milk from nonfresh, sick cows may have been included in the colostrum supply prior to this study. Misclassification of cows was eliminated through the colostrum management training, which emphasized proper identification of cows at their first and second milking and likely contributed to the significant increase in the IgG concentration of the first-milking pooled colostrum between the pretrial and trial periods. However, the training of the dairy's staff was provided by veterinarians who were present on the dairy daily for the first 3 weeks of the pretrial period and for the duration of the trial period. The level of training and oversight is not likely to be repeated by veterinarians working with producers, which may limit the effectiveness of similar colostrum management training in a field setting. In addition, this study was conducted on a single dairy and, hence, may not be generalizable to other dairies.

The study herd offered a unique opportunity to contrast results of the current trial to results from a previous trial that compared the effect of CDR or undifferentiated pooled colostrum on FPT, morbidity, and mortality in preweaning calves [12, 32]. Calves fed first-milking pooled colostrum alone in the current study had higher 24-hour serum IgG concentrations than calves fed pooled colostrum in the previous study (24.6 g/L and 7.50 g/L, resp.), whereas calves fed first-milking pooled colostrum followed by CDR had similar 24-hour serum IgG concentrations to calves fed CDR in the previous study (15.9 g/L and 15.15 g/L, resp.) on this dairy [12]. In addition, the risk of FPT in calves fed first-milking pooled colostrum alone was lower in the current study (0.0%, *n* = 58) compared to in the earlier study (70%, *n* = 269) [12]. Differences in the 24-hour serum IgG concentrations and FPT between the two studies may have been due to the increased amount of IgG fed to the calves in the current study or to differences in apparent efficiency of IgG absorption between the studies. In the current study, the IgG concentration in the first-milking pooled colostrum after the colostrum management training was higher (65.1 g/L) than previous pooled colostrum IgG concentrations measured from the same dairy in the earlier study (21.1 g/L) [12] but similar to the average IgG concentration in pooled colostrum from dairies in the Southwest region of the USA (64.3 g/L) [19]. The increase in the IgG concentration of the pooled colostrum in the current study is likely due to the improved colostrum collection by the dairy personnel following the colostrum management training. However, colostrum samples were not obtained prior to the initiation of the colostrum management training making it impossible to assess the direct effect of training.

The results of this study indicated that feeding calves either first-milking pooled colostrum alone or first- and second-milking pooled colostrum was associated with a significantly higher AEA than feeding calves first-milking pooled colostrum followed by CDR. These results are in contrast with the previous study conducted on this study dairy where the AEA of calves fed pooled colostrum (28.8%) was similar to the AEA of calves fed CDR (27.0%) [12]. However, the AEA of the calves fed first-milking pooled colostrum followed by CDR in the current study was similar to the AEA of calves fed CDR in the earlier study conducted on this dairy [12] but was lower than the AEA (35.5 to 38.8%) reported for calves fed CDR in other studies [8, 25]. Colostrum replacer products vary in the postcolostrum serum IgG concentrations which they can achieve in calves, despite providing similar amounts of IgG [20]. The use of different colostrum replacers among these studies may explain the variable AEA observed. Absorption of IgG across the intestinal epithelium is a saturable process [41, 42] and it is possible that other macromolecules present in the colostrum replacers may compete with the IgG absorption. Alternatively, differences in AEA observed among this and other studies may be due to differences in calf management practices among the studies, such as the amount of colostrum feedings provided, method of colostrum feeding, or time from birth to colostrum feeding that may have affected the calves' ability to absorb IgG from the colostrum [20, 43]. The results of this trial suggest that colostrum composition, in addition to volume and mass of IgG, is important in IgG absorption. Further research is needed to determine the effect of various macromolecules in colostrum and colostrum replacers on AEA and, in particular, the impact of supplementing maternal colostrum with colostrum replacers of varying composition.

Results of our study showed that dairies that feed high quality first-milking pooled colostrum can minimize or eliminate the risk of FPT in calves. Furthermore, during times of colostrum shortage, the colostrum supply can be extended by feeding 2 L of first-milking pooled colostrum combined with either 2 L of CDR or 2 L of second-milking pooled colostrum without significant differences in the risk of calves experiencing FPT and preweaning diarrhea or pneumonia.

## Figures and Tables

**Table 1:** Comparison of baseline characteristics among 3 treatments in a randomized field trial comparing the effects of feeding first-milking pooled colostrum alone or first-milking pooled colostrum followed by either a colostrum-derived replacer or second-milking pooled colostrum to newborn Holstein and Jersey calves.

Characteristic	Treatment 1*	Treatment 2*	Treatment 3*	*P* value
	*N* = 58	*N* = 54	*N* = 54	
Sex: *N* (%)				0.167^†^
Female	23 (39.7)	27 (50.0)	31 (57.4)
Male	35 (60.3)	27 (50.0)	23 (42.6)
Breed: *N* (%)				0.544^†^
Holstein	52 (89.7)	45 (83.3)	45 (83.3)
Jersey	6 (10.3)	9 (16.7)	9 (16.7)
Time to separation from dam: *N* (%)				0.912^†^
<1 hour	41 (70.7)	42 (77.8)	39 (72.2)
1–6 hours	15 (25.9)	11 (20.4)	14 (25.9)
6–9 hours	2 (3.4)	1 (1.9)	1 (1.9)
Time to colostrum feeding: *N* (%)				0.764^†^
<1 hour	4 (6.9)	3 (5.6)	4 (7.4)
1–6 hours	44 (75.9)	37 (68.5)	41 (75.9)
6–9 hours	10 (17.2)	14 (25.9)	9 (16.7)
Birth weight (kg):				0.750^§^
mean (SE^‡^)	39.02 (0.86)	38.68 (1.05)	37.99 (1.03)	
Precolostrum serum total protein (g/dL):				0.689^§^
mean (SE)	4.44 (0.04)	4.49 (0.04)	4.45 (0.06)	
Precolostrum serum IgG (g/L):				0.715^§^
mean (SE)	0.40 (0.05)	0.45 (0.06)	0.46 (0.06)	
First-milking colostrum IgG (g/L):				0.505^§^
mean (SE)	65.0 (2.16)	67.0 (2.01)	63.4 (2.35)	
Second-milking colostrum IgG (g/L):				—
mean (SE)	—	—	25.8 (1.81)	
Total IgG fed^||^ (g):				<0.001^§^
mean (SE)	255.9^a^ (8.87)	222.1^b^ (4.84)	172.2^c^ (4.75)	

*Treatment 1: 4L 1st-milking pooled colostrum, treatment 2: 2L 1st-milking pooled colostrum followed by 2L colostrum-derived replacer, and treatment 3: 2L 1st-milking pooled colostrum followed by 2L 2nd-milking pooled colostrum.

^†^Pearson chi-square or Fischer's exact test with 5% level of significance.

^‡^SE: standard error.

^§^One-way ANOVA using the Tukey multiple comparison procedure with 5% level of significance. Means without a common superscript are significantly different.

^||^Total IgG fed for treatment 2 estimated assuming 100 g IgG in colostrum-derived replacer.

**Table 2:** Effect of treatment on 24-hour serum total protein and IgG concentrations, apparent efficiency of IgG absorption, and passive transfer status of Holstein and Jersey calves in a randomized field trial comparing the effect of feeding either first-milking pooled colostrum alone or first-milking pooled colostrum followed by either a colostrum-derived replacer or second-milking pooled colostrum.

Item	Treatment 1*	Treatment 2*	Treatment 3*	*P* value
	*N* = 58	*N* = 54	*N* = 54	
24-hour serum total protein (g/dL):				
mean (SE^†^)	5.9^a^ (0.08)	5.2^b^ (0.07)	5.4^b^ (0.06)	<0.001^‡^
24-hour serum IgG (g/L):				
mean (SE)	24.6^a^ (1.06)	15.9^b^ (0.78)	18.3^b^ (0.66)	<0.001^‡^
Apparent efficiency of IgG absorption (%):				
mean (SE)	37.0^a^ (1.14)	26.3^b^ (1.01)	40.0^a^ (1.29)	<0.001^‡^
Failure of passive transfer: % (95% CI)				
IgG (<10 g/L)	0.0^a^ (—)	9.3^a^ (1.53, 16.99)	1.9^a^ (0, 5.45)	≥0.05^§^

*Treatment 1: 4L 1st-milking pooled colostrum, treatment 2: 2L 1st-milking pooled colostrum followed by 2L colostrum-derived replacer, and treatment 3: 2L 1st-milking pooled colostrum followed by 2L 2nd-milking pooled colostrum.

^†^SE: standard error.

^‡^One-way ANOVA using the Tukey multiple comparison procedure with 5% level of significance. Means without a common superscript are significantly different.

^§^Column proportions were compared using a *z*-test with the alpha adjusted for multiple comparisons using the Bonferroni method.

Means without a common superscript are significantly different at the 5% level.
